# Reactive Species-Activatable AIEgens for Biomedical Applications

**DOI:** 10.3390/bios12080646

**Published:** 2022-08-17

**Authors:** Xiaoying Kang, Yue Li, Shuai Yin, Wen Li, Ji Qi

**Affiliations:** 1 State Key Laboratory of Medicinal Chemical Biology, Frontiers Science Center for Cell Responses, Key Laboratory of Bioactive Materials, Ministry of Education, College of Life Sciences, Nankai University, Tianjin 300071, China; 2 Tianjin Key Laboratory of Biomedical Materials, Key Laboratory of Biomaterials and Nanotechnology for Cancer Immunotherapy, Institute of Biomedical Engineering, Chinese Academy of Medical Sciences and Peking Union Medical College, Tianjin 300192, China

**Keywords:** aggregation-induced emission, reactive oxygen nitrogen species, activatable probe, theranostics, fluorescence, photoacoustic, afterglow, bioimaging

## Abstract

Precision medicine requires highly sensitive and specific diagnostic strategies with high spatiotemporal resolution. Accurate detection and monitoring of endogenously generated biomarkers at the very early disease stage is of extensive importance for precise diagnosis and treatment. Aggregation-induced emission luminogens (AIEgens) have emerged as a new type of excellent optical agents, which show great promise for numerous biomedical applications. In this review, we highlight the recent advances of AIE-based probes for detecting reactive species (including reactive oxygen species (ROS), reactive nitrogen species (RNS), reactive sulfur species (RSS), and reactive carbonyl species (RCS)) and related biomedical applications. The molecular design strategies for increasing the sensitivity, tuning the response wavelength, and realizing afterglow imaging are summarized, and theranostic applications in reactive species-related major diseases such as cancer, inflammation, and vascular diseases are reviewed. The challenges and outlooks for the reactive species-activatable AIE systems for disease diagnostics and therapeutics are also discussed. This review aims to offer guidance for designing AIE-based specifically activatable optical agents for biomedical applications, as well as providing a comprehensive understanding about the structure–property application relationships. We hope it will inspire more interesting researches about reactive species-activatable probes and advance clinical translations.

## 1. Introduction

Precision medicine requires highly sensitive and specific diagnostic methods with high accuracy at the very early disease stage [1,2,3]. Some traditional imaging modalities such as ultrasound, computed tomography (CT), and magnetic resonance imaging (MRI) have been widely used in clinic [4,5,6]. However, most of them suffer from low sensitivity, and it is usually difficult to recognize tiny pathological changes when the lesion is small [7,8]. Optical imaging techniques such as fluorescence and photoacoustic imaging have significant advantages such as high sensitivity, real-time monitoring, noninvasive imaging, and portable instruments, which are very promising for disease diagnosis and therapy [9,10,11,12,13,14]. Fluorescence has been used for in vitro examination of diseased samples and in vivo image-guided tumor surgery clinically. However, due to interference from the strong light–tissue interaction (e.g., absorption, scattering, and reflection) and autofluorescence, the sensitivity of fluorescence is significantly reduced [15,16]. Therefore, the development of new imaging agents that could improve the therapeutic performance (e.g., recognition of disease-related markers) is highly desirable.

**Figure 1:**
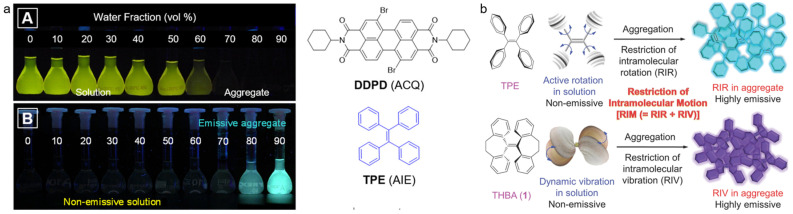
(**a**) Photographs of ACQ and AIE molecules in the mixture of water/THF with different water fractions under 365 nm of UV light irradiation (reproduced with the permission from Ref. [40]. Copyright 2018, American Chemical Society); (**b**) schematic illustration of RIM mechanism, including restriction of intramolecular rotation and restriction of intramolecular vibration. (Reproduced with the permission from Ref. [39]. Copyright 2014, WILEY-VCH Verlag GmbH & Co. KGaA, Weinheim).

Numerous materials have been used for optical imaging, for example, carbon nanomaterials, metal nanostructures, rare earth-doped nanoparticles (NPs), and organic materials [17,18,19,20,21]. Among them, organic compounds possess unique intrinsic merits including excellent reproducibility, specific chemical structures, and good biocompatibility [22,23,24,25,26]. Currently, small-molecule dyes, i.e., indocyanine green (ICG) and methylene blue (MB) have been approved by the Food and Drug Administration (FDA) for clinical use, highlighting the great clinical translation potential of organic optical materials [27,28,29]. Nevertheless, most conventional organic dyes are planar structures, which face the obstacle of aggregation-caused quenching (ACQ) effect in aggregate state due to strong intermolecular interactions (e.g., π-π stacking) [30,31]. The ACQ problem seriously hinders the applications of these hydrophobic molecules in a hydrophilic living environment. In 2001, Tang’s group first coined the the concept of aggregation-induced emission (AIE), representing a new type of optical materials that were weak or non-luminescent in dilute solution, but became highly emissive in aggregate form [32,33,34,35,36,37]. For AIE luminogens (AIEgens), the excited-state energy is consumed by the intensive intramolecular motion through non-radiative decay in solution, while the molecular motion is restricted in aggregate form, thus, the non-radiative pathway is closed and the radiative process is open (Figure 1) [38,39,40,41,42]. As a result, restriction of intramolecular motion (RIM) is considered to be the working principle of the AIE phenomenon, and a library of AIEgens with various properties have been developed [43,44,45,46]. AIEgens have been used in many areas such as optoelectronic devices, chemo/biosensing, and biological imaging [47,48,49,50]. In the biomedical field, AIEgens have shown excellent performance in organelle imaging, in vivo high-resolution imaging, disease theranostics, and activatable detection [51,52,53,54,55].

Excessive expression of various reactive species can lead to oxidative stress, which is known to cause DNA, protein, cell, and tissue damage, and affect signaling pathways [56,57,58]. These processes are closely associated with many diseases including inflammation, cancers, diabetes, and neurodegeneration diseases [59,60,61,62]. Thus, accurate detection and monitoring of these endogenously generated biomarkers is extensively important for precise disease diagnostics and therapeutics at an early stage [63,64,65]. According to their nature, reactive species can be divided into reactive oxygen species (ROS) including hydrogen peroxide (H_2_O_2_), hypochlorite/hypochlorous acid (HOCl/ClO^−^), hydroxyl radical (^•^OH), superoxide anion radical (O_2_^•−^), singlet oxygen (^1^O_2_), and peroxy radical (ROO^•^); reactive nitrogen species (RNS) including nitric oxide (NO), peroxynitrite (ONOO^−^), *S*-nitrosothiol (RSNO), and *S*-nitrosoglutathione (GSNO); reactive sulfur species (RSS) including hydrogen sulfide (H_2_S), thiyl radical (RS), thiol (RSH), *S*-nitrosothiol, sulfenic acid, and sulfite; reactive carbonyl species (RCS) including carbon monoxide (CO), formaldehyde (FA), glyoxal (GO), acrolein, and glucosone [66,67,68,69,70,71,72,73,74,75]. Reactive species have gained great interest from both fundamental biological scientists and clinical doctors, and more and more new phenomena about their functions have been discovered [76,77,78]. Numerous molecular probes for detecting ROS, RNS, RSS, and RCS have been exploited, focusing on understanding the physiological/pathological effects and disease theranostics [79,80,81,82,83,84]. Recently, the development of reactive species-responsive AIEgens has attracted considerable attention, which are advantageous for applications in the biomedical field [85,86,87,88].

Thanks to the salient merits of good stability, large Stokes shift, facile structure modification, and excellent sensitivity, AIEgens have emerged as a new type of potent probes for detecting various reactive species. Although there are many review papers that have focused on AIEgens [89,90,91,92,93,94], to the best of our knowledge, comprehensive summaries of reactive species-responsive AIEgens are very rare. In this review, we highlight the recent advances of AIEgen-based reactive species-activatable systems. The recent development of AIEgens for sensing reactive species such as ROS, RNS, RSS, and RCS are discussed. The molecular design strategies for increasing sensitivity, tuning the response wavelength, increasing the afterglow imaging efficiency, as well as different biomedical applications are reviewed. The challenges and outlooks for the reactive species-activatable AIE systems for biomedical applications are also discussed. This review aims to provide guidance for the development of activatable optical imaging agents with maximum disease biomarker recognition capability to improve the diagnostic and therapeutic outcomes of related biomedical applications. It provides a comprehensive understanding about the activatable molecular probe from molecular design, to probe property and biomedical applications, and thus, build the structure–property application relationships.

## 2. Detection of Reactive Oxygen Nitrogen Species

**Figure 2:**
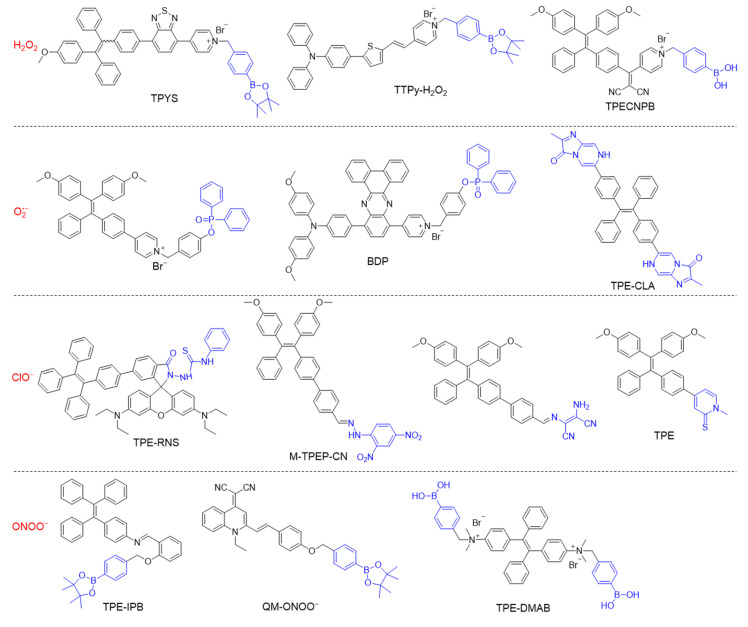
Chemical structures of different types of RONS-responsive molecules.

When designing a specific chemical/biological probe, a usually requisite is to synthesize molecules with specific recognition groups or moieties. The typical chemical structures of some reactive oxygen nitrogen species (RONS)-responsive AIEgens are listed in Figure 2. The boronate subunit is a popularly used building block for H_2_O_2_ sensors, as the boronate cage is nonfluorescent and the conversion of arylboronates to phenols results in turn-on emission [95,96,97]. The deprotonated H_2_O_2_ is a potent nucleophile, which can attack the boron center to generate a labile borate species that hydrolyses to the corresponding phenol [98]. For O_2_^•−^ detection, the diphenyl phosphinyl group can be introduced into an organic compound, in which the fluorescence is strongly quenched at first, and obvious turn-on fluorescent signal is realized in the presence of O_2_^•−^ [99,100]. The oxidative properties of ClO^−^ can be utilized to destroy C=C or C=N bonds rapidly, therefor, the conjugation of fluorescence quencher through C=C or C=N bonds has turned out to be an efficient strategy to construct ClO^−^ probes [101,102]. Some arylboronate groups, diphenylphosphinate groups, and nitrophenyloxoacetamide moieties have been employed as the response substitutes for ONOO^−^ detection [103,104,105]. The tunability of molecular structure will alter the photophysical properties and biomedical applications as well.

**Figure 3:**
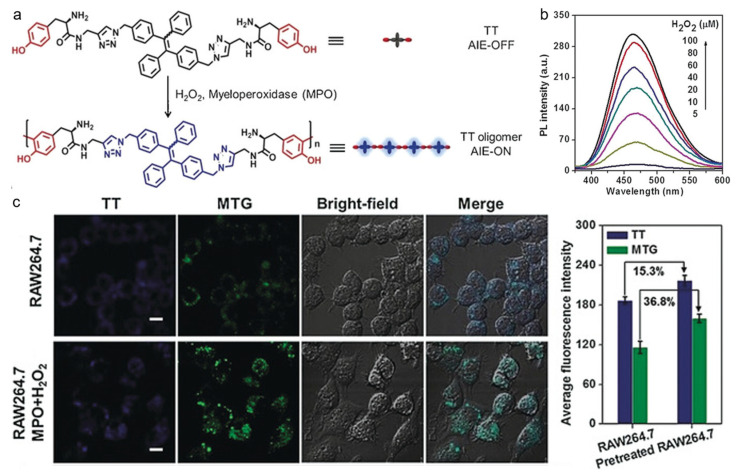
(**a**) Peroxidase-catalyzed polymerization in the presence of H_2_O_2_; (**b**) PL spectra of TT with the treatment of different concentrations of H_2_O_2_; (**c**) CLSM images and corresponding fluorescence intensity of RAW264.7 cells pretreated without and with MPO and H_2_O_2_ incubating with TT. Scale bars: 20 µm. (Reproduced with the permission from Ref. [113]. Copyright 2018, Wiley-VCH Verlag GmbH & Co. KgaA, Weinheim).

H_2_O_2_ is an overexpressed molecule in many serious diseases, and thus, it is regarded as a pivotal biomarker for some biological processes and disease diagnoses [106,107,108]. A variety of H_2_O_2_-activatable probes have been exploited based on AIEgens, which exhibit excellent performance for both in vitro and in vivo applications [109,110,111,112]. Xia and Lou et al. developed a H_2_O_2_-responsive AIEgen for peroxidase-mediated selective imaging and inhibition of inflammatory cells [113]. As shown in Figure 3, the probe consisted of a TPE core and two tyrosine (Tyr) moieties, which could undergo enzyme-catalyzed dityrosine formation in the presence of peroxidase and H_2_O_2_. By conjugating two hydrophilic Tyr groups, the hydrophobic TPE molecule became hydrophilic TT, which showed weak fluorescence in aqueous solution due to the excited-state energy consumption via intense molecular motion. As a result, the H_2_O_2_-responsive and myeloperoxidase (MPO)-mediated TT self-assembly enabled turn-on fluorescence, which could be used for selectively imaging and inhibiting inflammatory cells containing overexpressed H_2_O_2_ and MPO. The AIE process could be activated through dityrosine linkage-induced hydrophobic aggregates formation, which helped to distinguish between inflammatory and normal cells. Additionally, the in situ formation of TT aggregates could inhibit RAW264.7 cell growth through inducing mitochondria damage and cell apoptosis.

**Figure 4:**
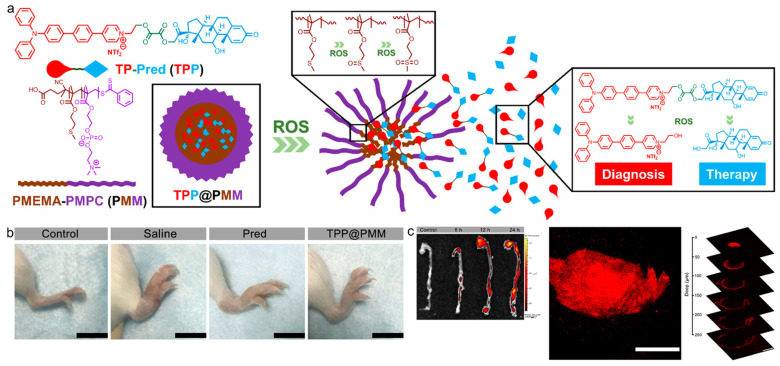
(**a**) Schematic of the theranostic nanoplatform with serial ROS response; (**b**) photographs of the hind limbs from arthritic mice with different treatments; (**c**) two-photon fluorescence imaging of aortas, atherosclerotic plaques, and plaques. Scale bars: 200 μm. (Reproduced with the permission from Ref. [114]. Copyright 2020, American Chemical Society).

Wang and Li et al. reported a ROS-responsive theranostic nanoplatform for accurate diagnosis and therapy of inflammation diseases [114]. As depicted in Figure 4, a two-photon AIEgen (TP) was conjugated with the widely used anti-inflammatory glucocorticoid, prednisolone (Pred) with the ROS-sensitive linkage to afford the compound TPP. Then, the TPP was encapsulated with an amphiphilic block copolymer PMPC−PMEMA (PMM) to give polymeric micelles (TPP@PMM). Noteworthy, the PMEMA part served as the hydrophobic block in the NPs formation, which could be oxidized in response to ROS to yield the hydrophilic sulphone product. The ROS-triggered hydrophobic-to-hydrophilic conversion was able to realize ROS-mediated drug delivery at an inflammatory site. This shell-core dual ROS-responsive nanoplatform was used in three different inflammatory murine models including acute lung injury, atherosclerosis, and arthritis. The deep-penetration two-photon fluorescence diagnosis and efficient serial ROS sensitive anti-inflammation could be used for both acute and chronic inflammation theranostics. Two-photon imaging with the AIEgen helped to provide unambiguous delineation of inflammatory tissue with minimum autofluorescence interference. Moreover, TPP@PMM also possessed excellent anti-inflammatory effect that reduced the inflammatory response and decreased inflammatory cytokines expression.

**Figure 5:**
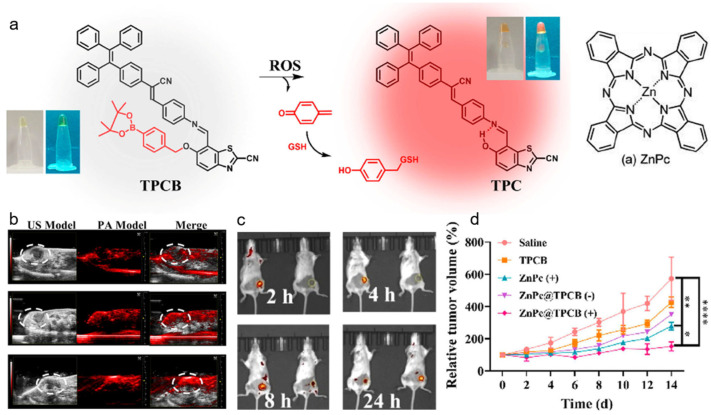
(**a**) Chemical structure and the ROS-activatable process of TPCB probe; (**b**) in vivo ultrasound and PA and (**c**) fluorescence imaging of 4T1 tumor-bearing mice after injecting the nanoprobe; (**d**) tumor growth curves of 4T1 tumor-bearing mice with different treatments. * *p* < 0.05, ** *p* < 0.01, **** *p* < 0.001. (Reproduced with the permission from Ref. [120]. Copyright 2021, American Chemical Society).

Photoacoustic (PA) imaging is an emerging biomedical imaging modality that originates from the thermoelastic expansions of light-absorption chromophores generating ultrasound signal [115,116,117]. PA imaging possesses the merits of good penetration depth and excellent spatial resolution, which has complementary advantages with fluorescence technique, thus, integration of PA imaging and fluorescence could greatly improve the diagnostic outcome [118,119]. Qu, Feng, and coworkers reported an activatable system for dual-modal imaging-guided PDT and self-reporting therapeutic process [120]. As displayed in Figure 5, the nanoplatform was composed of an AIEgen with ROS-recognition phenylboronate moiety and a photosensitizer ZnPc. Due to the ACQ effect, ZnPc in NPs was non-emissive with low PDT efficiency and strong PA signals, which could monitor the tumors’ locations with reduced phototoxicity. When ZnPc was gradually released from the NPs, its fluorescence and ROS generation property could be recovered, which was capable of reporting the release process. Upon light irradiation, the ROS generated by ZnPc could induce cell apoptosis and activate the AIE-based ROS probe, serving as an indicator of ROS production. Moreover, as the side product of AIEgens, quinone methide (QM) could deplete GSH in cancer cells, which enhanced the PDT effect. In vitro and in vivo experiments both revealed that this intelligent platform enabled highly efficient and accurate PDT of tumors. The activatable theranostic nanoprobe could eliminate the phototoxicity of conventional photosensitizer and provide clinicians with guidance about detailed disease information, as well as therapeutic process and outcome, rendering great promise for individual therapy and precision medicine.

**Figure 6:**
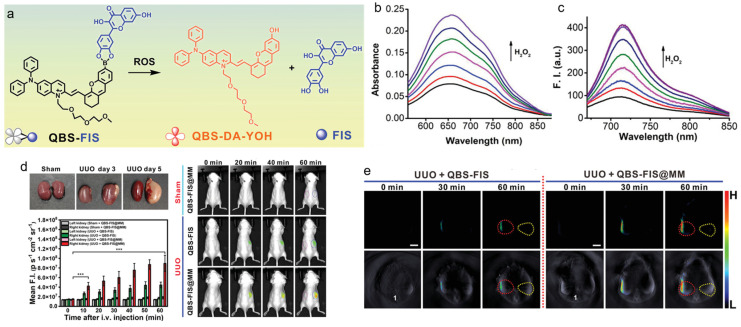
(**a**) Chemical structure and ROS response of the theranostic probe; (**b**) absorption and (**c**) PL spectra of QBS-FIS with the treatment of different concentrations of H_2_O_2_; (**d**) fluorescence and (**e**) PA imaging of the sham-surgery and unilateral ureteral obstruction (UUO) mice with different treatments. *** *p* ≤ 0.001. (Reproduced with the permission from Ref. [121]. Copyright 2021, Wiley-VCH GmbH).

Wu, Tian, and Zeng et al. reported a ROS-activatable multifunctional nanosystem for liver and kidney inflammation diagnosis and therapy through modulating inflammatory pathways [121]. As presented in Figure 6, an AIEgen was linked with a Nrf2 activator fisetin through boronate bond, in which the fluorescence would be quenched and the boronate bond could be cleaved by ROS. This probe was co-encapsulated with a NF-kB inhibitor thalidomide, and macrophage cell membrane was employed as the coating to ensure effective target and accumulation in the inflammatory sites. In the lipopolysaccharides (LPS)/*D*-galactosamine (*D*-GalN)-induced acute liver injury/inflammation mouse model, the nanoprobe could actively target the inflammatory site and the boronate bonds could be cleaved by the overexpressed ROS, therefore, activating the near-infrared (NIR) fluorescence and PA signal for precisely imaging liver/kidney inflammatory diseases and the released drugs were able to treat acute liver inflammation through activation of the Nrf2 pathway and suppression of the NF-κB signaling pathway with moderate suppression of NLRP3 inflammasome formation. The fluorescence and PA imaging were capable of monitoring the therapeutic process as well.

**Figure 7:**
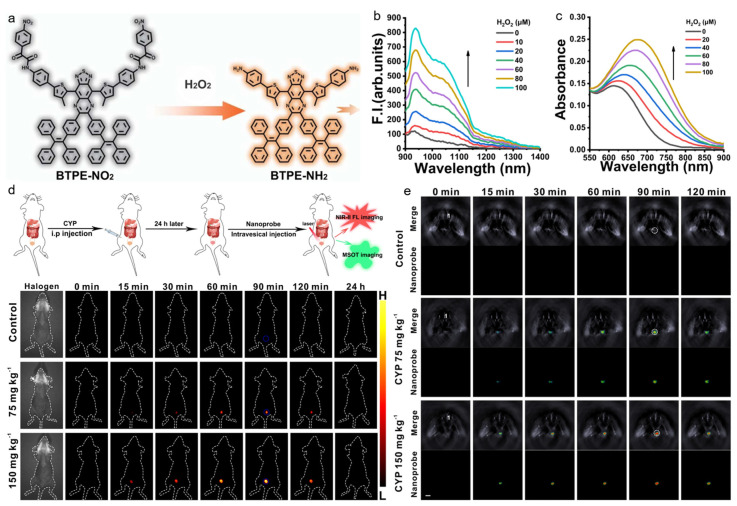
(**a**) The AIE probe for H_2_O_2_ detection; (**b**) PL and (**c**) absorption spectra of BTPE-NO_2_ with the treatment of different concentrations of H_2_O_2_; (**d**) NIR-II fluorescence and (**e**) PA imaging of interstitial cystitis mice with different treatments at designed time points after administration. (Reproduced with the permission from Ref. [127]. Copyright 2021, The Authors).

Although a conventional NIR region (NIR-I, 700–900 nm) is considered to be a transparent biological window, it is still very difficult to realize high-resolution in vivo imaging. The recently emerging second NIR (NIR-II, 1000–1700 nm) window exhibits great promise for bioapplications as it possesses significantly reduced light–tissue interaction and enables large-depth and high-resolution imaging in a living body [122,123,124,125,126]. Zhao and Wu et al. reported on a H_2_O_2_-activatable AIE nanoprobe for sensitive disease diagnosis via NIR-II fluorescence and PA imaging [127]. As displayed in Figure 7, the low-bandgap D-A compound consisted of two TPE groups that would result in AIE feature and increased conjugation, and two nitrophenyloxoacetamide moieties that could be cleaved in the presence of H_2_O_2_. The probe was nonfluorescent because the strong electron-withdrawing nitrophenyl group quenched the emission, but significant NIR-II fluorescence could be observed after the H_2_O_2_-inducing nitrobenzene cleavage. In the presence of H_2_O_2_, the maximal absorption of AIE nanoprobe shifted from 615 nm to 680 nm, and pronounced NIR-II fluorescence with a peak at 938 nm and a shoulder peak at 1028 nm was also obtained, which enabled NIR-II fluorescence and PA imaging of H_2_O_2_-overexpressed diseases. After intravesically injecting into the interstitial cystitis mice, the AIE probe realized H_2_O_2_-activatable NIR-II fluorescence in the bladder, with 3D PA imaging to locate the bladder inflammation. In vivo experiments in trazodone-induced liver injury mice and liver ischemia-reperfusion injury mice also showed excellent diagnostic performance, indicating that the nanoprobe was a robust tool for detecting and imaging H_2_O_2_-related diseases with NIR-II fluorescence and PA imaging.

**Figure 8:**
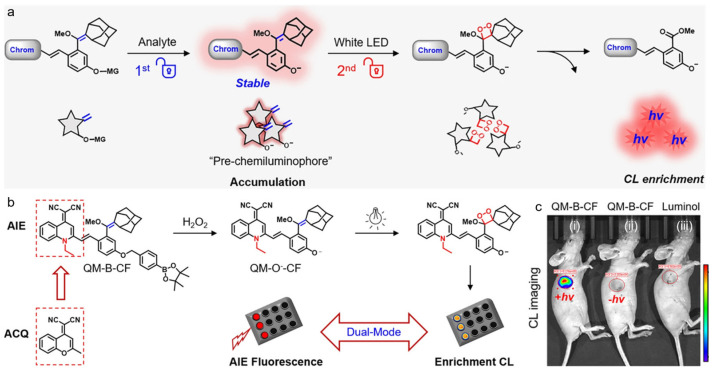
(**a**) Schematic of the dual-lock probe triggered sequentially by analyte and light for CL imaging; (**b**) the reaction processes of AIE-based dual-lock probe QM-B-CF; (**c**) in vivo imaging of 4T1 xenograft tumor-bearing mice after intratumor injection of QM-B-CF or luminol. (Reproduced with the permission from Ref. [134]. Copyright 2020, Wiley-VCH Verlag GmbH & Co. KGaA, Weinheim).

Chemiluminescence (CL) is a revolutionized imaging technique for in vivo monitoring of biospecies in which the signal contrast and sensitivity can be significantly increased since the light emission is initiated by a chemical reaction with minimal autofluorescence interference [128,129,130,131]. However, most traditional CL emitters are dependent on the generation of unstable and short-lived emitting species, making the photons release uncontrollable, and dynamic biological imaging difficult [132,133]. Guo and coworkers reported on a sequential dual-lock photoactivatable chemiluminescent AIE probe for bright optical imaging [134]. As shown in Figure 8, for the first lock, the caging group was triggered and removed by the analyte, generating pre-chemiluminophores with twisting intramolecular charge transfer (TICT) property; for the second lock, the electron-rich double bond was activated by light for in situ generation of 1,2-dioxetane, accompanied with enhanced CL signal. As compared with traditional dioxetane-based one-lock CL probe, this type of dual-lock probe containing dicyanomethylene-4*H*-pyran (DCM) fluorophore displayed nearly 10-fold higher signal. The authors further constructed a probe based on AIEgen of quinoline-malononitrile (QM) unit, which displayed remarkably bright CL signal. After intratumorally injecting into xenograft 4T1 tumor-bearing mice, the nanoprobe showed gradually increased tumor microenvironment H_2_O_2_-activatable CL signal and realized an ultra-high S/N ratio 74 times higher than the background, in which the CL intensity was around 66-fold higher than that of typically used luminol emitter.

**Figure 9:**
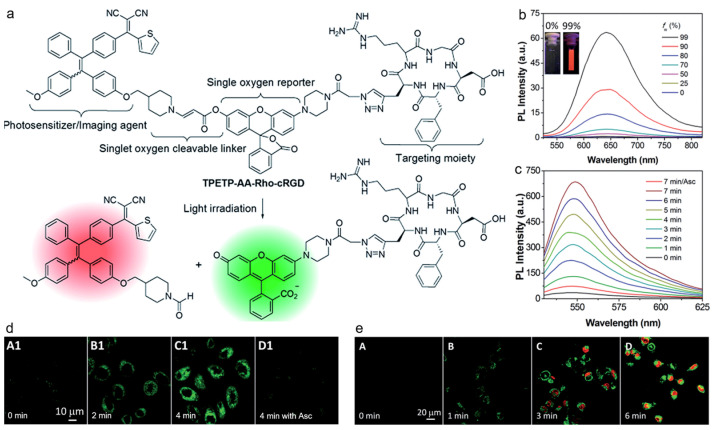
(**a**) Schematic illustration of TPETP-AA-RhocRGD for self-reporting ^1^O_2_ detection; (**b**) photoluminescence of TPETP in DMSO/water mixture with different water fractions; (**c**) photoluminescence spectra of the probe under light irradiation at different time points; (**d**,**e**) CLSM images of MDA-MB-231 cells incubated with the probe under light irradiation at different time points as indicated. (Reproduced with the permission from Ref. [138]. Copyright 2016, The Royal Society of Chemistry).

Photodynamic therapy (PDT) is a clinically used method for treating some cancers, which is based on a light-triggered photosensitizer to generate ROS, especially the highly toxic ^1^O_2_ [135,136,137]. To monitor the in situ generation of ^1^O_2_ during PDT in real time is of comparative significance for tumor therapy and reduced side effects. Liu and coworkers reported on a self-reporting AIE probe for real-time monitoring of ^1^O_2_ generation and targeted PDT [138]. As depicted in Figure 9, the probe was constructed by conjugating a red emissive AIEgen and a rhodol dye with green fluorescence through ^1^O_2_-cleavable aminoacrylate (AA) linker. The probe TPETP-AA-Rho-cRGD emitted red fluorescence at first, whereas strong green fluorescence from rhodol could be observed upon image-guided light irradiation as the AA linker was cleaved by the photogenerated ^1^O_2_, which could be used for real-time and in situ monitoring of ^1^O_2_ production during PDT. After incubating with MDA-MB-231 cells followed by light irradiation for different periods of time, the green fluorescence from the probe intensified with time as more ^1^O_2_ was produced, and there was nearly no fluorescence in the cells treated with ^1^O_2_ scavenger ascorbic acid (Asc). These results indicated that the probe was capable of efficiently reporting the generated ^1^O_2_ concentration_._ The green fluorescence from the probe matched well with the red fluorescence from propidium iodide (PI), which demonstrated that the probe could be used to report ^1^O_2_ generation and predict the therapeutic effect in real time.

**Figure 10:**
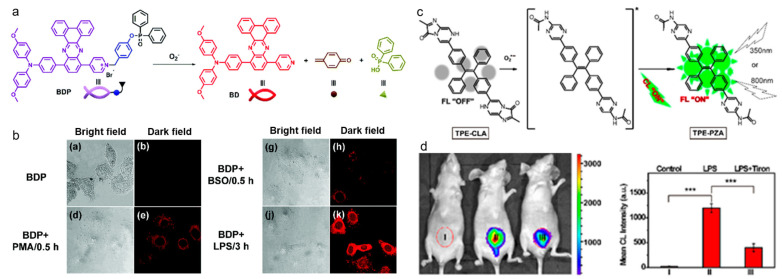
(**a**) Chemical structure of BDP and the O_2_^•−^-activatable process; (**b**) CLSM imaging of HepG2 cells with different treatments (reproduced with the permission from Ref. [141]. Copyright 2018, The Royal Society of Chemistry); (**c**) chemical structure and the O_2_^•−^-response mechanism; (**d**) in vivo CL images and corresponding imaging intensity of LPS-induced inflammation in mice. *** *p* < 0.001. (Reproduced with the permission from Ref. [142]. Copyright 2017, American Chemical Society).

O_2_^•−^ is regarded as the primary ROS in the living body, the overproduction of which causes oxidative stress and disruption of the redox balance [139,140]. Thereby, precise detection of entogenous O_2_^•−^ is of critical significance for understanding related diseases. Hua and coworkers reported on a NIR emission AIE probe for O_2_^•−^ detection (Figure 10a,b) [141]. They synthesized a D-A-type compound with methoxy-substituted triphenylamine and dibenz[a,c]-phenazine as the D and A moieties, respectively, which showed maximal emission in the NIR region of longer than 700 nm and a large Stokes shift, and the diphenyl-phosphinyl group was linked as it could be cleaved by O_2_^•−^. The BDP probe exhibited weak emission, whereas the fluorescence was intensified in the presence of O_2_^•−^, which enabled good sensitivity and selectivity. The probe was first incubated with HepG2 cells, which were then treated with exogenous O_2_^•−^ producer such as LPS, *L*-buthionine sulfoximine (BSO), and phorbol-12-myristate-13-acetate (PMA), generating very strong NIR fluorescence. Tang’s group developed an AIE probe for endogenous O_2_^•−^ detection with turn-on fluorescence/CL imaging [142]. As shown in Figure 10c, the probe was synthesized by conjugating TPE with imidazopyrazinone (CLA), which was a well-established recognition group to O_2_^•−^. In the presence of O_2_^•−^, the CLA unit was oxidized to form a dioxetanone that decomposed to generate a singlet-excited amide, which then decayed to the ground state with concomitant CL emission and fluorescence as well. TPE-CLA was a highly sensitive probe to O_2_^•−^ with low detection limits of 0.21 nM for fluorescence and 0.38 nM for CL. In the Raw264.7 cells pretreated with PBS (control), Tiron (a scavenger of O_2_^•−^), and PMA (a stimulator of O_2_^•−^), two-photon imaging of TPE-CLA with 800 nm excitation showed weak fluorescence in the control group, negative fluorescence in the Tiron group, and very bright fluorescence in the PMA group, which suggested that the probe was capable of detecting the endogenously stimulated O_2_^•−^ and also capable of imaging native O_2_^•−^ in living cells. In the LPS-induced acute inflammation model, TPE-CLA displayed rather strong CL signal, while the luminescence was quenched by mixing with Tiron, which demonstrated that the probe could be used as a specific O_2_^•−^ biosensor in vivo.

**Figure 11:**
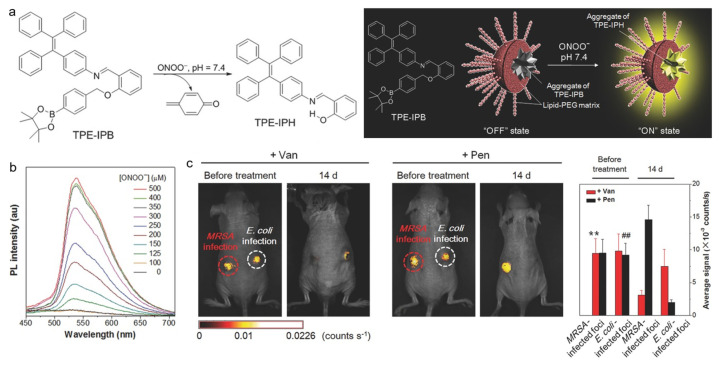
(**a**) TPE-IPB probe for ONOO^−^ detection with turn-on fluorescence; (**b**) PL spectra of TPE-IPB with the treatment of different concentrations of ONOO^−^; (**c**) in vivo fluorescence images and the corresponding fluorescence intensity of the mice infected with *MRSA* at left and *E. coli* at right before and after vancomycin and penicillin treatment. ** *p* < 0.01, in comparison between *MRSA*-infected foci before and after vancomycin treatment for 14 d; ## *p* < 0.01, in comparison between *E. coli*-infected foci before and after penicillin treatment for 14 d. (Reproduced with the permission from Ref. [146]. Copyright 2016, Wiley-VCH Verlag GmbH & Co. KGaA, Weinheim).

The excessive expression of ONOO^−^ is an important feature of many major diseases such as cancer, diabetes, cardiopathy, Alzheimer’s disease, and acute and chronic inflammation [143,144,145]. Consequently, the specific detection of ONOO^−^ is momentous for diagnosing these diseases and monitoring the therapeutic process. Tang and Ding et al. reported on an AIE-based probe for ONOO^−^ detection and related inflammation imaging in living mice [146]. As shown in Figure 11, the phenylboronic ester in TPE-IPB was cleaved when treating with ONOO^−^, and the hydrogen bond formed in the product TPE-IPH led to bright fluorescence with a peak at 538 nm. The probe exhibited good selectivity toward ONOO^−^ over other species such as H_2_O_2_, O_2_^•−^, ^•^OH, ROO^•^, ClO^−^, and tert-butyl hydroperoxide (TBHP). After intravenous injection into living mice, the AIE nanoprobe showed selective turn-on fluorescence in the inflammatory region with elevated ONOO^−^ production. In addition, the probe could also help to precisely and noninvasively monitor the in vivo therapeutic efficacy of antiinflammatory agents. After subcutaneously inoculating *MRSA* and *Escherichia coli* (*E. coli*) at different sides of nude mice, the inflammation-bearing mouse model with different infections was built. The AIE probe could clearly distinguish the inflammation-bearing mice with vancomycin for treating *MRSA*-caused infections or penicillin for treating *E. coli*-caused infections.

**Figure 12:**
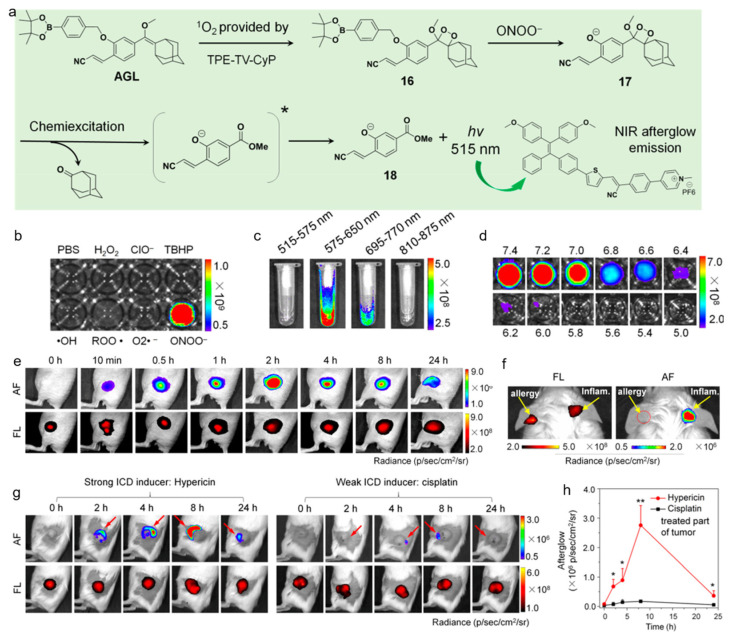
(**a**) Chemical structures and working mechanism of the ONOO^−^ and pH dual-response afterglow luminescence; (**b**) selectivity of the nanoprobe with various ROS treatments; afterglow intensity of the nanoprobe (**c**) in different spectral regions and (**d**) different pH environments; (**e**) fluorescence and afterglow images of the acute inflammation after injecting the preirradiated nanoprobe at different time points; (**f**) fluorescence and afterglow images of the mouse with allergic left ear and inflammatory right ear; (**g**) fluorescence and afterglow images of the 4T1 tumor-bearing mice at different time points after receiving PDT with hypericin or cisplatin treatment and (**h**) corresponding afterglow intensity. * *p* < 0.05, ** *p* < 0.01. (Reproduced with the permission from Ref. [147]. Copyright 2022, American Chemical Society).

Recently, Ding’s group reported on an AIEgen with ONOO^−^ and pH dual-responsive afterglow luminescence for neutrophil-involved diseases applications [147]. As depicted in Figure 12, an AIEgen and a Schaap’s dioxetane-based agent was mixed into one system to realize NIR afterglow luminescence, which could be triggered by both ONOO^−^ and surrounding pH value. The working principle of this nanosystem was as follows: The AIEgen could produce ^1^O_2_ under light exposure, which oxidized the enol ether structure to four-membered 1,2-dioxetane with no phenylborate protection; the disease site-overexpressed ONOO^−^ could cleave the phenylborate group to afford unstable dioxetane intermediate, and then emit persistent green luminescence; the energy transfer between the excited-state four-membered 1,2-dioxetane compound and AIEgen led to bright NIR afterglow luminescence for specific afterglow imaging of disease sites. The activatable nanoprobe possessed 553-fold enhancement in emission intensity upon the treatment of ONOO^−^, while it showed little response to other species such as H_2_O_2_, O_2_^•−^, ^•^OH, ROO^•^, ClO^−^, and TBHP. Noteworthy, th—ONOO^−^-activated afterglow signal possessed a pH-dependent manner, as the intensity was very high at pH 7.0−7.4 and intensely decreased in low pH. As both ONOO^−^ generation and acid environment were closely related to the inflammation processes, the ONOO^−^ and pH dual-response characteristics allowed for precise inflammation imaging. The NIR afterglow luminescence of the nanoplatform could last for 14 days and achieve a high SBR of 29 with coverage of 10 mm chicken breast issues. In the LPS-induced acute skin inflammation of BALB/c mice, the nanoprobe dissolved in 5× PBS (pH 7.4) exhibite—ONOO^−^-activated afterglow luminescence and the intensity reached a maximum with a SBR of 461.3 at about 2 h. In the mouse model with ovalbumin-induced allergic skin disease in the left ear and LPS-induced acute inflammation in the right ear, very strong activated afterglow luminescence from the preirradiated probe was observed in the LPS-treated ear skin, but the afterglow signal was still in the “off” state in the allergic ear, which was due to the significant difference in infiltration of neutrophils and accumulation of ONOO^−^ between the two disease models. Immunogenic cell death (ICD) represents a promising cell demise mode with a signature of immunostimulatory damage-associated molecular patterns (DAMPs) emission and transformation from an environmental cold tumor to a hot tumor, during which neutrophils as the first innate immune responders are recruited into the tumor bed and promote the proliferation of CD8^+^ T cells in antitumor immunity. This probe was able to report the levels of infiltrating neutrophils and ONOO^−^ generation in vivo, being beneficial for screening the ICD drugs in a fast and real-time manner.

## 3. Detection of Gasotransmitters

**Figure 13:**
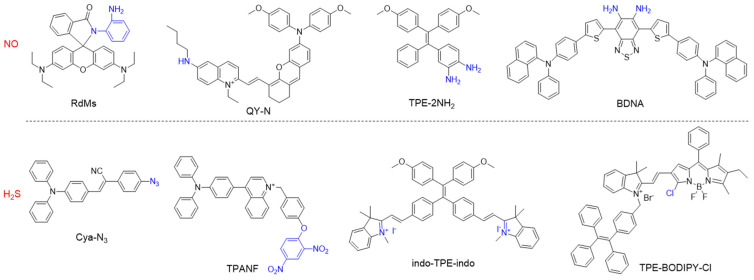
Chemical structures of different types of gasotransmitter-responsive molecular probes.

Small gaseous molecules including NO, CO, and H_2_S, function as important signal transmitters in living systems as they are associated with many biological functions and major diseases [148,149,150,151]. NO is a neutral diatomic free radical that is produced from *L*-arginine by NO synthase (NOSs) isoforms such as neuronal NOS (nNOS), inducible NOS (iNOS), and endothelial NOS (eNOS) [152,153]. CO is the second gasotransmitter that is generated as a byproduct of haem cleavage by two distinct haem oxygenases [154]. H_2_S is predominantly formed from Cys or its derivatives by the enzymes cystathionine β-synthase and cystathionine γ-lyase [155]. All these gasotransmitters play vital roles in vasorelaxation and inflammatory responses, thus, numerous molecular probes have been developed for precise monitoring of related diseases [156,157,158]. For example, the *o*-diamino aromatic moiety is a recognition group for NO, and the cyclization reaction of *o*-diamine with NO produces a triazole moiety, which alters the electronic property and conjugation nature [159,160,161]. For H_2_S detection, the popularly used approaches include reduction of azides into amines and nucleophilic addition of H_2_S to the electrophilic group [162,163]. Some representative AIEgens for sensing gasotransmitters are listed Figure 13, which show great potential for applications in biological imaging and disease diagnosis.

**Figure 14:**
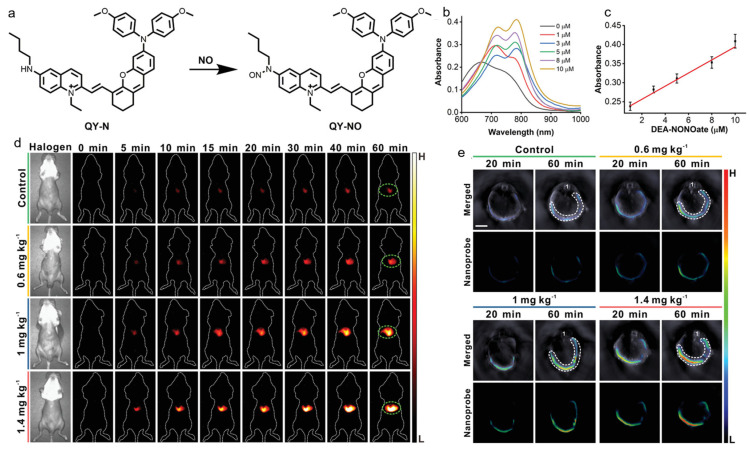
(**a**) The response of QY-N towards NO; (**b**) absorption spectra and (**c**) intensity at 780 nm of QY-N with the treatment of different concentrations of DEA·NONOate; (**d**) NIR-II fluorescence and (**e**) PA imaging of liver injury mice intravenously injected with the nanoprobe QY-N at different time points. (Reproduced with the permission from Ref. [164]. Copyright 2021, Wiley-VCH GmbH).

Wu’s group developed a NO-activatable AIEgen for precisely diagnosing herbal medicine-induced liver injury with NIR-II fluorescence and PA imaging [164]. As presented in Figure 14, they designed and synthesized a D-*π*-A-type probe (QY-N) consisting of an electron-rich bismethoxyphenyl-amine-containing dihydroxanthene group and an electron-deficient quinolinium moiety. The linking of electron-donating butylamine to the quinolinium group weakened the electron-accepting capability, and thus, quenched the fluorescence, and butylamine also served as a NO-responsive group based on the *N*-nitrosation reaction of aromatic secondary amine. In the presence of NO, the electron-donating butylamine was transformed into an electron-withdrawing butyl-*N*-nitroso group, which resulted in a bathochromic shift of absorption in the range of 700–850 nm for PA imaging, and boosted NIR-II fluorescence at 910–1110 nm. The AIE probe was able to detect and assess the severity of herbal medicine-induced liver injury in vivo in a high-contrast manner for significantly enhanced NIR-II fluorescence and PA signals via reacting with the overexpressed NO at a disease site. In addition, the probe was also capable of monitoring the rehabilitation of liver injury during the treatment process.

**Figure 15:**
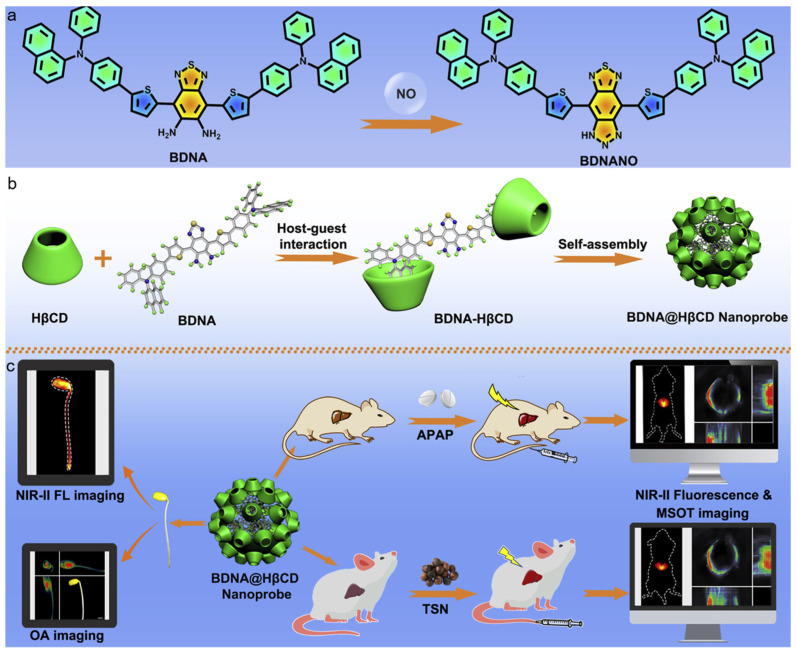
(**a**) The mechanism of BDNA for NO detection; (**b**) the formation of host–guest supramolecular complex BNDA–HβCD and the nanoprobe; (**c**) the BNDA@HβCD nanoprobe for in vivo NIR-II fluorescence and PA imaging of liver injury and detecting NO in soybean sprouts. (Reproduced with the permission from Ref. [165]. Copyright 2021, The Authors).

Recently, Wu and Zeng et al. developed an activatable nanoprobe with AIE feature for detecting NO with NIR-II fluorescence and PA imaging [165]. As displayed in Figure 15, the *o*-phenylenediamino group was incorporated as the core because it could react with NO, and two phenylnaphthalenamine moieties were conjugated to function as the electron donors and endow AIE characteristic. Then, the FDA-approved 2-hydroxypropyl-b-cyclodextrin (HβCD) was incorporated through the formation of a host–guest supramolecular complex to ensure good water dispersibility and biocompatibility without sacrificing NO responsivity. The resultant BNDA–HβCD complex was able to self-assemble into nanoaggregates in aqueous media, which displayed very weak absorption and fluorescence in the NIR spectral region. While, in the presence of NO, the *o*-phenylenediamino moiety reacted with NO to yield a triazole group, which greatly enhanced the electron-withdrawing capability and afforded strong absorption at 650–850 nm for PA imaging and fluorescence at 900–1100 nm for NIR-II imaging. By reacting with the disease-overexpressed NO, the nanoprobe was successfully applied for detecting and imaging liver injuries and monitoring the therapeutic outcome through activatable NIR-II fluorescence and PA imaging. Moreover, the nanoprobe was also capable of detecting and tracking endogenous NO in soybean sprouts.

**Figure 16:**
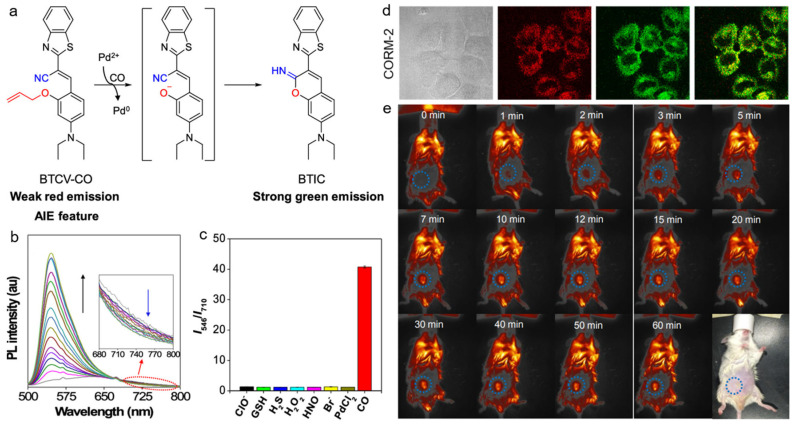
(**a**) The mechanism of AIE-based probe for CO detection; (**b**) PL spectra of BTCV-CO probe incubated with different concentrations of CORM-2; (**c**) PL intensity ratios (*I*_546_/*I*_710_) of BTCV-CO with the treatment of various biomolecules; (**d**) CL images of MCF-7 cells incubated with the probe; (**e**) in vivo fluorescence imaging of CO with the probe. (Reproduced with the permission from Ref. [169]. Copyright 2019, American Chemical Society).

High concentrations of CO (>35 ppm) cause high toxicity to living bodies, while this type of gas with low concentration is recognized as a biological regulator [166,167]. For example, CO can modulate inflammatory responses, promote neovascular growth, and prevent vascular dysfunction and tissue ischemia [168]. Therefore, the sensitive detection of CO is critically important for monitoring the related biological processes. Wang and coworkers developed an AIEgen-based probe (BTCV-CO) for CO detection and visualization [169]. As displayed in Figure 16, the allyl group in the BTCV-CO probe could be removed via CO treatment ([Ru(CO)_3_Cl_2_]_2_ (CORM-2) was used as the CO donator) to generate the phenolate intermediate, which underwent rapid cyclization and afforded the benzithiazolyl iminocoumarin (BTIC) product with bright fluorescence. During this process, a new fluorescence peak appeared at 546 nm, and the emission at long wavelength declined. As a result, the ratiometric response of *I*_546_/*I*_710_ displayed 39-fold enhancement, and the detection limit of CO was calculated to be as low as 30.8 nM. BTCV-CO also exhibited good selectivity as it showed no response toward other interfering species such as ClO^−^, GSH, H_2_S, H_2_O_2_, HNO, and Br^−^. The BTCV-CO probe could image CO sensitively in both CORM-2-treated cells and living mice.

**Figure 17:**
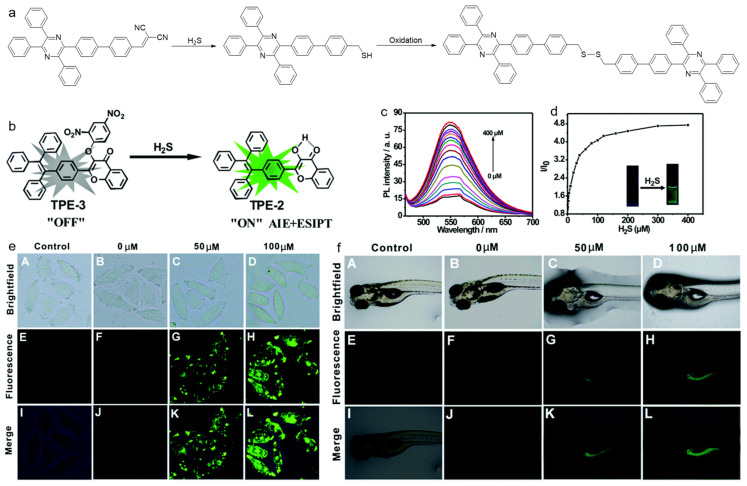
(**a**) The proposed reaction process of TPP-PDCV for detecting H_2_S; (**b**) the AIE probe for H_2_S detection with turn-on fluorescence; (**c**) PL spectra and (**d**) the corresponding PL intensity ratio of the AIE probe treated with different concentrations of H_2_S; Fluorescence imaging of (**e**) HeLa cells and (**f**) zebrafish larvae treated with different concentrations of H_2_S. (Reproduced with the permission from Ref. [171]. Copyright 2016, The Royal Society of Chemistry and the Chinese Chemical Society).

Tang and coworkers reported an AIEgen probe for detecting H_2_S [170]. As described in Figure 17a, the D-A compound was synthesized using tetraphenylpyrazine (TPP) as D, and malonitrile group as A. In the presence of H_2_S, the malonitrile was oxidized to a thiol intermediate, which underwent self-coupling to produce a dimer. During this process, the D–A interaction was destroyed, which resulted in a blue shift in the emission wavelength. The probe could be used for specific and sensitive H_2_S detection. Wu and Zeng et al. developed a fluorescent probe with both AIE and excited-state intramolecular proton transfer (ESIPT) characteristics for H_2_S detection (Figure 17b–f) [171]. Due to the strong electron-withdrawing property of the nitrobenzene group, the fluorescence of the probe was remarkably quenched. While in the presence of H_2_S, the nitrophenyl moiety was cleaved, and ESIPT feature occurred, thereby, the fluorescence intensified. The probe was formulated into water-dispersible NPs, which showed fast response and excellent selectivity toward H_2_S over many other reactive species. In vitro experiments in HeLa cells revealed that the nanoprobe was capable of detecting the exogenous and endogenous H_2_S. For zebrafish pretreated with the nanoprobe, there was no fluorescence in the untreated fish, while strong green fluorescence was observed in the fish incubated with NaHS-containing (NaHS was used as the H_2_S donor) media. Except for the detection of various ROS, RNS, RSS, and RCS, AIEgens have also been utilized for monitoring other disease-associated biomarkers such as pH, alkaline phosphatase (ALP), glutathione (GSH), β-galactosidase, hypoxia, etc. [172,173,174,175,176]. Due to limited space, we do not discuss these in this review.

## 4. Summary

In this review, we highlight the recent advances of AIE-based probes for detecting reactive species (including ROS, RNS, RSS, and RCS) and related biomedical applications. The molecular design approaches for constructing activatable AIEgens are summarized, and their applications in monitoring major diseases such as cancer, inflammation, and vascular diseases are also discussed. These types of probes turn out to be highly efficient for sensitive detection and precise disease theranostics. Future development can be focused on several aspects. First, the absorption and PL wavelengths of the reported AIE probes are relatively short, in which the unsatisfied penetration depth would limit in vivo applications. A bathochromic shift of the response region to a long-wavelength NIR-II region by tuning molecular structure would be beneficial for real applications. Second, the response of molecular probe in aggregate usually decreases as compared with the solution state, therefore, there is still some room to improve the response time and selectivity of AIE probes. For example, to design an AIE probe that is soluble in water at first and forms an aggregate after reacting with specific reactive species, would increase the sensitivity, and also realize turn-on fluorescence by making full use of AIE feature. Third, the afterglow-based detection imaging has a great advantage for bioimaging as it does not need external light excitation. Therefore, the activatable-afterglow AIE probe is favorable for high-contrast biosensing and diagnosis. Moreover, although reactive species play a key role in modulating many biological processes and diseases, and a number of probes have been developed, the real applications are still very rare. For future clinical transformation, biocompatibility as well as improved detection specificity and sensitivity should be carefully considered. Reactive species are important biomarkers for many diseases, yet they may not be the specific criterion for pathological changes and their abnormity may not be related to a specific disease as well. Thus, the combination of reactive species-based imaging and other diagnostic approaches would increase the disease theranostic precision. This review aims to offer guidance for designing AIE-based specifically activatable optical agents for biomedical applications, as well as provide a comprehensive understanding about the structure–property application relationships. We hope it will inspire more interesting research on reactive species-activatable probes and advance the clinical translations.
